# Fetal aortic valvuloplasty: first report of two cases from Saudi Arabia

**DOI:** 10.1186/s13019-020-01195-y

**Published:** 2020-06-22

**Authors:** Merna Atiyah, Ahmed Kurdi, Osama Al Tuwaijry, Atif Al Sahari, Maha Al Rakaf, Inas Babic, Fahad Al Habshan, Zohair Alhalees, Khalid Al Najashi

**Affiliations:** 1 Pediatric Cardiology Department, Prince Sultan Cardiac Center, Prince Sultan Military Medical City, As Sulimaniyah, Riyadh, 12233 Saudi Arabia; 2 Obstetrics & Gynecology Department, Prince Sultan Military Medical City, Riyadh, Saudi Arabia; 3 King Abdul-Aziz Cardiac Center, National Guard Hospital, Riyadh, Saudi Arabia; 4 Department of Cardiac Surgery, King Faisal Specialist Hospital & Research Center, Riyadh, Saudi Arabia

**Keywords:** Aortic valve stenosis, Fetal aortic Valvuloplasty, Fetal echocardiography

## Abstract

**Background:**

Fetal aortic stenosis may progress to hypoplastic left heart syndrome (HLHS), which carries a poor prognosis. We report two infants with fetal aortic stenosis successfully treated with fetal aortic valvuloplasty (FAV) using balloon dilatation.

**Case presentation:**

Of five fetuses with aortic stenosis fulfilling the FAV criteria of severe aortic stenosis with a left ventricular length Z-score of ≥ − 2, retrograde flow in the transverse aortic arch, left-to-right flow across the foramen ovale, monophasic mitral inflow, and significant left ventricular dysfunction, we obtained permission for FAV in two fetuses. FAV was performed successfully under echocardiographic guidance using balloon dilatation. Both fetuses survived to birth. During FAV, mild pericardial effusion developed when introducing the stylet needle in the second fetus, and this resolved within 48 h. No intraprocedural complications occurred in the first patient, and no maternal complications occurred. The first infant underwent the Ross procedure after birth and is currently 7 years old and doing well. The second patient underwent aortic and mitral valve repair with endocardial fibroelastosis resection approximately 2 weeks after birth, which temporarily addressed the mitral valve stenosis; high doses of inotropes were subsequently required. The infant died of sepsis at 2 months of age.

**Conclusion:**

FAV using balloon dilatation to treat fetal aortic stenosis was successful in our two patients, with subsequent neonatal biventricular repair resulting in long-term survival in one patient and death secondary to sepsis in the second patient.

## Background

Hypoplastic left heart syndrome (HLHS) occurs in up to 3% of infants with congenital heart disease and is considered the main cause of death in this population [1]. The syndrome is a spectrum of left heart growth abnormalities in which the left heart is unable to support cardiac output. The underlying mechanisms are controversial, although there is strong evidence for a genetic component in a small subset of patients. This is consistent with a multifactorial etiology. In some cases, HLHS is established early in pregnancy [1]. In other cases, however, HLHS develops secondary to severe aortic stenosis (AS) in mid-gestation. The left ventricle (LV) is usually initially normal, but as gestation progresses, the LV may become dilated with impaired function. This further decreases the potential growth of the LV, leading to HLHS [2, 3].

Several reports have described the performance of FAV for early relief of the obstruction at the aortic valve (AOV). The goal of this procedure is to modify the natural history of the disease, which might preserve left heart growth and prevent HLHS [4–9]. In one study, biventricular circulation was achieved after birth in about 59% of cases, with a per-procedural pregnancy loss rate of 4% [10].

In this study, we share our initial experience of initiating an FAV program in two patients diagnosed with critical AS, aiming to relieve the obstruction of the AOV before the development of irreversible damage to the LV.

## Case presentation

### Indication

Since we began our fetal intervention program in 2009, 10 fetal cases have been referred for treatment of critical AS. Five of these cases fulfilled the following criteria for FAV: severe AS with a left ventricular length Z-score of ≥ − 2, retrograde flow in the transverse aortic arch, left-to-right flow across the foramen ovale (FOV), a monophasic mitral inflow pattern, and significant left ventricular dysfunction [2]. Only three families consented to the fetal intervention. One fetus had hydrops fetalis and died before the procedure. In the remaining two cases, the parents were counseled about the nature of the procedures, and informed consent was obtained.

### Technique

**Fig. 1:**
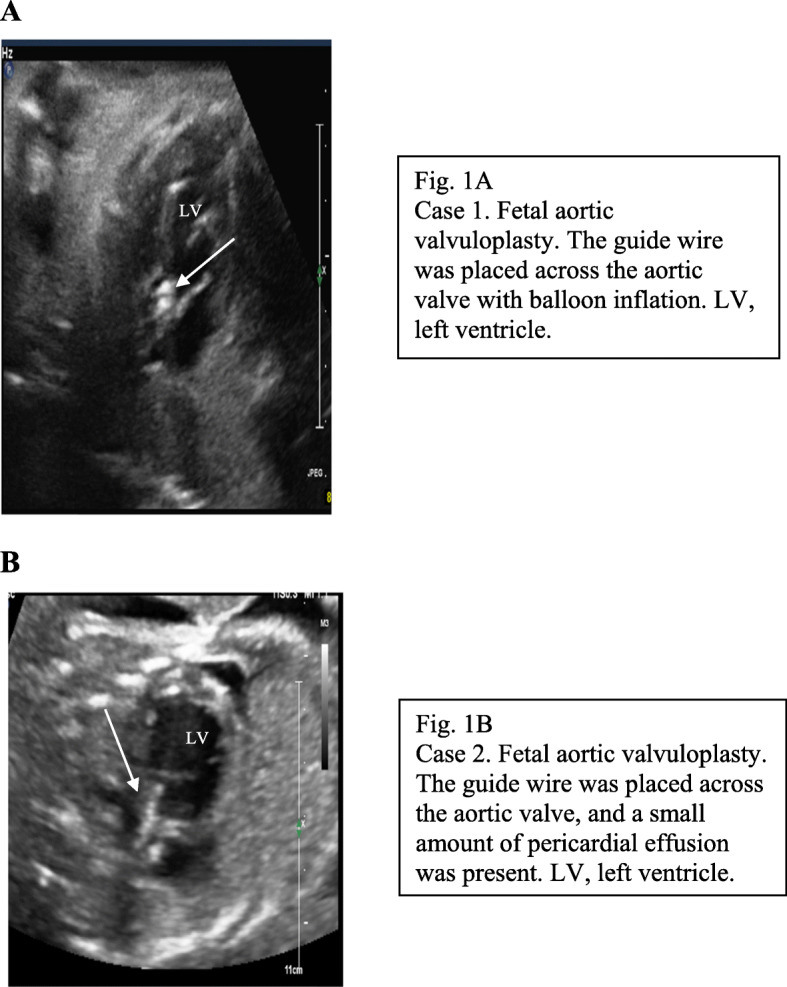
**a** Case 1. Fetal aortic valvuloplasty. The guide wire was placed across the aortic valve with balloon inflation. LV, left ventricle. **b** Case 2. Fetal aortic valvuloplasty.The guide wire was placed across the aortic valve,and a small amount of pericardial effusion was present. LV, left ventricle

An ideal fetal position was obtained (left side of fetal chest wall anteriorly oriented, left outflow tract parallel to intended cannula course). Under continuous echocardiography guidance (iU22 in Case 1 and EPIQ 7C in Case 2; Philips Healthcare, Andover, MA, USA), a 15-cm-long 18-gauge cannula with a stylet needle was introduced into the maternal abdomen and directed from the fetal thorax to left ventricular apex, aiming toward the AOV (Fig. 1a, b). After advancing the cannula until just beneath the AOV, the stylet needle was removed, and a 0.014-in. guide wire with a coronary dilatation catheter was introduced across the AOV. A 4.0-mm balloon was used, and the balloon was inflated twice by a pressure gauge in both cases.

**Fig. 2:**
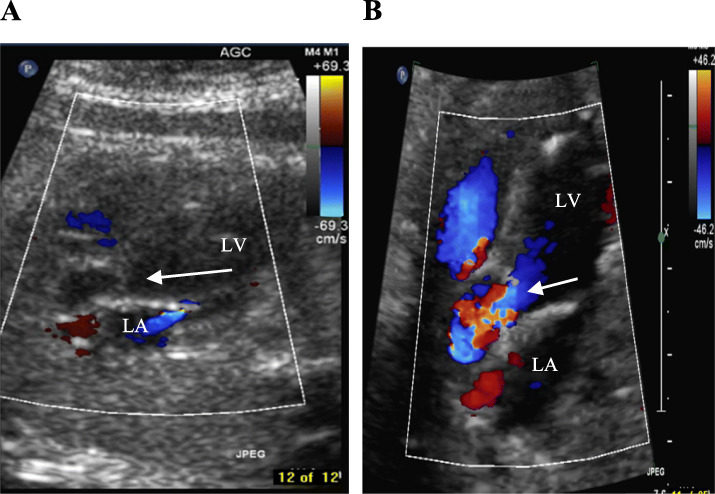
**a** Before the intervention, no forward flow (arrow) was present through the left ventricular outflow tract and mitral regurgitation was observed by color Doppler. **b** Immediately after the balloon dilation, echocardiography demonstrated increased forward flow across the aortic valve and disappearance of the mitral regurgitation. LV, left ventricle; LA, left atrium

After withdrawal of the balloon, we noted immediate antegrade flow across the AOV (Fig. 2a, b) and a moderate degree of aortic regurgitation (AR) by color Doppler in both cases (Fig. 3), indicating technical success of the procedures.

**Figure:**
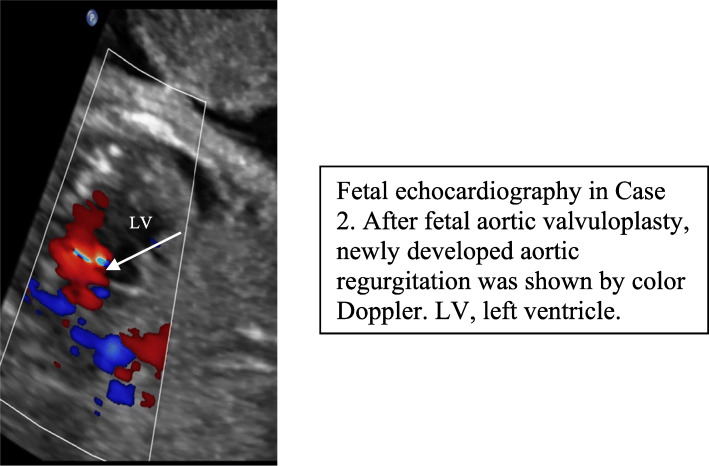
Fig. 3

No complications occurred in Case 1 (Video 1, **pre and post AV balloon dilatation)**. Mild pericardial effusion developed during needle introduction in Case 2 (Video 2, **pre and post AV balloon dilatation)**, but resolved spontaneously during the next 48 h. No maternal complications occurred in either case.


**Additional file 1: Video 1.** Case 1 pre and post AV balloon dilatation.



**Additional file 2: Video 2.** Case 2 pre and post AV balloon dilatation.


#### Case 1

A 25-year-old pregnant woman was referred at 31 weeks of gestation. She had no significant medical history. Detailed fetal echocardiography showed cardiomegaly with dilation and poor contraction of the LV. The AOV was immobile with no forward flow in the left outflow tract (Fig. 2a), and blood filled the aorta by reverse flow from the ductus arteriosus. The aortic annulus diameter was 3.8 mm. We also observed left-to-right flow across the FOV, a monophasic mitral inflow pattern, mild mitral regurgitation (MR), and a left-sided aortic arch. The fetus also had mild ascites and pleural effusion, suggesting hydrops and heart failure secondary to severe critical AS. No extracardiac anomalies were observed.

The fetal orientation was favorable for intervention from the start such that a direct approach as described above was smoothly performed from the apex and was successful on the first attempt. Serial follow-up fetal echocardiography assessments showed improvement of left ventricular function, improvement of aortic flow, disappearance of MR, and reversal of the shunt across the FOV. The fetus was delivered by normal vaginal delivery with a birth weight of 2.7 kg. Postnatal echocardiography showed unicuspid thickening and a stenotic AOV with a mean pressure of 58 mmHg and no AR. The neonatal Ross procedure was performed after postnatal balloon dilatation had resulted in residual severe AV stenosis and moderate AR. At the time of this writing, the child was 7 years old and doing well.

#### Case 2

**Fig. 4:**
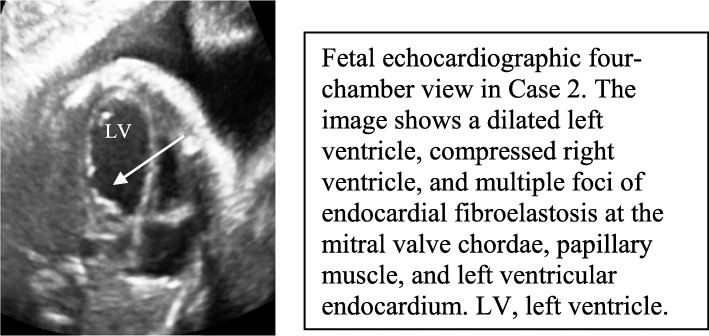
Fetal echocardiographic four-chamber view in Case 2. The image shows a dilated left ventricle, compressed right ventricle, and multiple foci of endocardial fibroelastosis at the mitral valve chordae, papillary muscle, and left ventricular endocardium. LV, left ventricle

A 31-year-old pregnant woman was referred at 28 weeks of gestation. Her previous child had died of a neurological disorder in the first year of life. Detailed fetal echocardiography showed that the LV was dilated with severely decreased ventricular function and that multiple sites of endocardial fibroelastosis (EFE) were present in the LV endocardium and MV chordae and papillary muscles (Fig. 4). The AOV was thickened, dysplastic, and immobile with no forward flow. Retrograde flow was seen from the ductus arteriosus, the aortic annulus diameter was 3.2 mm, left-to-right flow was present across the FOV, and a monophasic inflow pattern was observed. Trivial MR was present with no sign of fetal hydrops. No extracardiac anomalies were present.

The fetal position was initially unfavorable with the spine facing anteriorly, but spontaneous fetal movement resulted in a more suitable position about 2 h later. The technical success of the procedure appeared to be confirmed by an improvement in aortic flow on Doppler echocardiography (Fig. 3) (the ventricular function did not improve, although the AR disappeared). A second attempt to perform FAV was discussed; however, some forward flow was seen through the AOV, prompting a wait-and-see approach. The FOV continued to exhibit left-to-right shunting, and there was no further regression of the LV growth.

The male fetus was delivered at 37 weeks of gestation by Caesarean section with a birth weight 2.9 kg. Postnatal echocardiographic assessment showed a dysplastic bicuspid and stenotic AOV with forward flow and measuring 4 mm. Mild MR was present, and the MV measured 8 mm. The infant had severely impaired left ventricular function and multiple foci of EFE. He did not require prostaglandin.

The initial strategy was to promote biventricular circulation. Clear forward flow was present in the AOV, so the infant was started on high-dose inotrope therapy to improve cardiac function. The left ventricular systolic function gradually began to improve in the second week of life, and with this improvement in function, the MV inflow showed significant stenosis and significant EFE involved the whole MV apparatus. The infant underwent aortic and MV repair and EFE resection. Immediately postoperatively, the MV stenosis decreased; however, few days later the left atrial pressure began to gradually increase again with increase in MV stenosis and elevated pulmonary artery pressure. This could be explained mitral valve disease. The patient continue high ventilator setting due to pulmonary oedema. The patient eventually died of sepsis at 2 months of age.

## Discussion

Several mechanisms been proposed for the development of hypoplasia or borderline size of the left heart structures. The most widely proposed mechanism suggests that this clinical entity starts as severe or critical AS and progresses to an increased left ventricular afterload, resulting in decreased systolic and diastolic function. Reverse shunting across the FOV decreases the volume of blood crossing the MV, thus decreasing the further potential for growth of the left heart structures [2, 3].

Chronic elevation of ventricular pressure in the setting of AS may not only affect the growth of the left heart structure but may also damage the fetal myocardium, thus affecting ventricular systolic and diastolic function [11, 12]. Diastolic dysfunction can result from increased stiffness of the ventricle, which replaces the normal myocardial tissues with fibrotic collagen called EFE [13–15].

In Case 2, the fetus had significant EFE affecting the left ventricular endocardium and MV apparatus (grade 3 according to McElhinney et al. [16]). However, even after FOV, the left ventricular function did not improve until the postnatal period; where inotropic support initiated and ventricle function start to improve, the pressure gradient across the MV start to show up .. The combination of aortic stenosis, mitral stenosis and significant EFE in a neonate, even with an adequate LV size, is a risk factor for a poor outcome [16–18].

This report describes our growing experience with first-attempt FAV to establish a foundation for future clinical practice of FAV in Saudi Arabia. Both FAV procedures in this report were technically successful as indicated by inflation of the balloon across the valves and improvement in anterograde color flow and/or new AR, as previously defined [19, 20].

Previously published data revealed high success rates of FAV (range, 70–81%) and improved rates of achieving biventricular circulation (range, 43–67%) [10, 20, 21]. However, patient selection remains challenging, especially in patients with extensive EFE affecting the valves and myocardium. More data are required to assess the impact of FAV on achieving biventricular circulation.

## Conclusion

Our initial experience described in this report will help to establish the future clinical practice of FAV. Careful selection of patients and centralization of experiences in medical institutions with high levels of technical skill may improve the perinatal outcomes of fetal intervention in the region. .

## Data Availability

Not applicable (medical records and images are available in the hospital).

## Abbreviations

HLHS: Hypoplastic Left Heart Syndrome
FAV: Fetal Aortic Valvuloplasty
AS: Aortic Stenosis
LV: Left Ventricle
AOV: Aortic Valve
FOV: Foramen Ovale
AR: Aortic Regurgitation
MR: Mitral Regurgitation
EFE: Endocardial Fibroelastosis
